# Surpassing the 10% efficiency milestone for 1-cm^2^ all-polymer solar cells

**DOI:** 10.1038/s41467-019-12132-6

**Published:** 2019-09-10

**Authors:** Baobing Fan, Wenkai Zhong, Lei Ying, Difei Zhang, Meijing Li, Yanrui Lin, Ruoxi Xia, Feng Liu, Hin-Lap Yip, Ning Li, Yuguang Ma, Christoph J. Brabec, Fei Huang, Yong Cao

**Affiliations:** 1 Institute of Polymer Optoelectronic Materials and Devices, State Key Laboratory of Luminescent Materials and Devices, South China University of Technology, Guangzhou, 510640 P. R. China; 2 Department of Physics and Astronomy, Shanghai Jiao Tong University, Shanghai, 200240 P. R. China; 3 Institute of Materials for Electronics and Energy Technology (i-MEET), Friedrich-Alexander-Universität Erlangen-Nürnberg, Martensstr. 7, 91058 Erlangen, Germany; 4 Helmholtz Institute Erlangen-Nürnberg for Renewable Energy (HI ERN), Immerwahrstr. 2, 91058 Erlangen, Germany; 5 National Engineering Research Center for Advanced Polymer Processing Technology, Zhengzhou University, 450002 Zhengzhou, China

**Keywords:** Materials science, Optics and photonics

## Abstract

Naphthalenediimide-based n-type polymeric semiconductors are extensively used for constructing high-performance all-polymer solar cells (all-PSCs). For such all-polymer systems, charge recombination can be reduced by using thinner active layers, yet suffering insufficient near-infrared light harvesting from the polymeric acceptor. Conversely, increasing the layer thickness overcomes the light harvesting issue, but at the cost of severe charge recombination effects. Here we demonstrate that to manage light propagation within all-PSCs, a thick bulk-heterojunction film of approximately 350 nm is needed to effectively enhance photo-harvesting in the near-infrared region. To overcome the severe charge recombination in such a thick film, a non-halogenic additive is used to induce a well-ordered micro-structure that inherently suppresses recombination loss. The combined strategies of light management and delicate morphology optimization lead to a promising efficiency over 10% for thick-film all-PSCs with active area of 1 cm^2^, showing great promise for future large-scale production and application of all-PSCs.

## Abstract (web summary)

Thick film is preferred for sufficient light absorption and tolerant processing but it easily spoils the charge transport in the all-polymer solar cells. Here Fan *et al*. reconcile by tuning the film morphology and deliver high efficiency of 10% in 1 cm^2^ area devices.

## Introduction

All-polymer solar cells (all-PSCs) have attracted much interests for their unique advantages, e.g., superior ambient stability, enhanced mechanical flexibility, and processing versatility^1–3^. The critical issues that hamper the development of all-PSCs include the limited light-harvesting capability of the photoactive layer, low charge carrier mobility, formation of unfavorable film morphology, and so forth^4,5^. Although a number of valid strategies have been developed to control the domain size of all-polymer blends, the photovoltaic performance of all-PSCs was still restricted by the limited categories of n-type polymers. Therefore, the selection of an appropriate n-type polymer is of great importance for realizing highly efficient all-PSCs. To date, several notable families of n-type polymers, such as cyanated polyphenylenevinylene, benzothiadiazole, boron-nitrogen coordination units, perylene diimide, naphthalene diimide (NDI), and so forth, have been utilized for constructing all-PSCs^6–10^. Among them, NDI-bsased n-type polymers, especially the polymer N2200^11^ and their derivatives, have been intensively investigated for their broad optical absorption band, high electron mobility, and excellent chemical stability. The record efficiencies of all-PSCs have been realized in systems incorporating N2200 as the acceptor component^12–14^.

However, one drawback of the N2200 derivatives is their strong crystallization tendency, making nanoscale phase-separation in the bulk-heterojunction (BHJ) layer hard to be acquired^15^. Despite this issue can be addressed by various strategies including chemical modification and device processing^16–19^, much less efforts are concerned on large-area devices. The scientific progress stimulated us to address the issues affecting all-PSCs, i.e., insufficient near infrared (NIR) absorption by thin active layers, severe charge recombination in thick active layers, and relatively low efficiency of large-area devices. Currently, the performance of thick-film and/or large-area devices still lags behind the requirements for practical applications, with a moderate efficiency of around 9% for devices with active layer thickness of about 300 nm and around 7% for device with active area up to 1 cm^2^ (Supplementary Fig. 1)^20,21^.

In this contribution, we carefully investigate the optical and recombination behaviors in thick-film all-polymer devices. We emphasize the importance of thick active layer for enhancing the NIR-light harvesting of N2200-based acceptors. Through optical simulation on the basis of the transfer matrix formalism, we observe notably different optical field distributions in active layers with different thickness, such that a thick active layer of about 350 nm is obligatory to promote the *J*_SC_ to a theoretical value of 20 mA cm^−2^. Combined with a morphology-optimization strategy using the solvent additive dibenzylether, we demonstrate an experimental *J*_SC_ approaching 18 mA cm^−2^ along with a high fill factor (FF) over 72%, resulting in a high efficiency of 10.4%, for a 380-nm-thick all-PSC with a device area of 0.05 cm^2^. More importantly, the efficiency can be maintained above 10% for large-area devices with an active area of 1 cm^2^, thereby further mitigating the gap relative to organic solar cells based on molecular acceptors.

## Results

### Optical properties

**Fig. 1:**
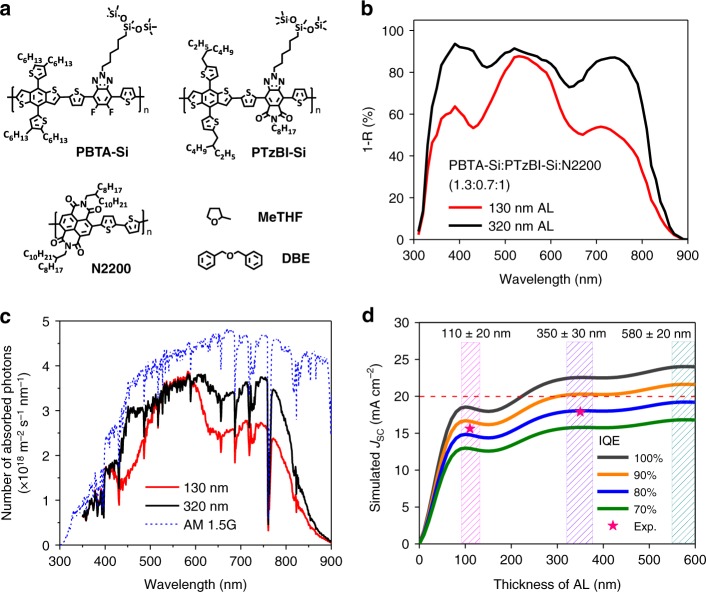
Absorption profiles and optical simulations. **a** Chemical structure of active layer components and solvents. **b** Absorption profiles for PBTA-Si:PTzBI-Si:N2200 (1.3:0.7:1) blend films with different active layer thickness, determined by reflection (double-pass) mode. **c** Number of absorbed photons versus wavelength for 1.3:0.7:1-cells with active layer thickness of 130 nm and 320 nm, simulated by transfer matrix formalism. **d** Simulated *J*_SC_ versus active layer thickness based on constant IQE, with the red stars represent *J*_SC_ calculated from experimental IQE; the dashed line and patterned areas are guides to the eyes

The all-polymer system investigated here was based on the blend of PBTA-Si:PTzBI-Si:N2200 (chemical structures shown in Fig. 1a), which showed complementary absorption spectra (Supplementary Fig. 2). The initial screen of the blend ratio of this composite gives the optimal ratio of 1.3:0.7:1, for which the device exhibited the optimal performance with the highest FF (Supplementary Figs. 3 and 4, Supplementary Tables 1 and 2). To reveal the real absorption of device with optimal blend ratio, we included the electrode reflection (R) with the parasitic absorption subtracted^22^. The relative intensity of the N2200 absorption increased substantially for both 130-nm and 320-nm films (Fig. 1b). The 320-nm-thick film showed a flat absorption band corresponding to a high EQE response (Supplementary Fig. 4b). The optical simulations (Supplementary Figs. 5 and 6) based on the transfer matrix formalism^23,24^ revealed that increasing the thickness of active layer to 320 nm led to obviously increased number of photons at a wavelength range of 600–900 nm (Fig. 1c). As N2200 is the only NIR photon absorber in this system, it is rational to correlate the sharply increased *J*_SC_ in thick film device to N2200. Based on the number of photons harvested in the active layer, the simulated *J*_SC_ versus thickness of the active layer was plotted under different internal quantum efficiency (IQE) scales, where IQE was assumed to be wavelength-independent. As shown in Fig. 1d, there are three *J*_SC_ plateaus (the patterned regions) as the active layer thickness increases, located at the thickness ranges of 110 ± 20, 350 ± 30, and 580 ± 20 nm, respectively. It is worth noting that this all-polymer system has a potential *J*_SC_ exceeding 20 mA cm^−2^ when the IQE reaches 90%.

### Thick-film devices

Two classic all-polymer systems, PCE10:N2200^25^ and PBDB-T:N2200^26^, also showed enhanced NIR absorption by N2200 with increased film thickness, resulting in broader EQE spectra and higher integrated current density (Supplementary Figs. 7 and 8). For devices based on PCE10:N2200 and PBDB-T:N2200 (with an active area of 0.05 cm^2^), increasing the active layer thickness to around 300 nm caused a sharp decrease of FF compared with those having active layers on the 100 nm scale (Supplementary Table 3). This trend was also realized in the pristine PBTA-Si:PTzBI-Si:N2200 (1.3:0.7:1) film (Supplementary Fig. 8c). Although the thin-film (130 nm) device showed a relatively high FF exceeding 75%, increasing the thickness of active layer to 320 nm lowered the FF to <65% (Table 1).

**Table 1:** Photovoltaic parameters for all-PSCs based on PBTA-Si:PTzBI-Si:N2200 (1.3:0.7:1)

Area (cm^2^)	Thickness (nm)	Solvent additive	*V*_OC_ (V)	*J*_SC_, _EQE_^d^ (mA cm^−2^)	FF (%)	PCE^f^ (%)
0.05^a^	130	/	0.85	14.24	75.3	9.1
	320	/	0.83	16.30	64.6	8.7
0.05^a^	130	DBE^c^	0.83	14.07	80.0	9.3
	380	DBE^c^	0.82	17.52	72.1	10.4
1.0^b^	130	DBE^c^	0.85	15.05^e^	76.3	9.8
	350	DBE^c^	0.84	17.76^e^	66.8	10.0

The errors are defined as the standard deviation

^a^Aperture with an area of 0.04 cm^2^ was used

^b^Aperture with an area of 0.9062 cm^2^ was used

^c^The volume ratio of DBE is 0.1%

^d^*J*_SC_, _EQE_ is defined as the calculated current density from EQE spectra

^e^The average of current densities obtained from EQEs with light-spots concentrating on three locations of 1.0 cm^2^-cell

^f^The efficiency is calculated by, PCE = *V*_OC_·*J*_SC_, _EQE_·FF

**Fig. 2:**
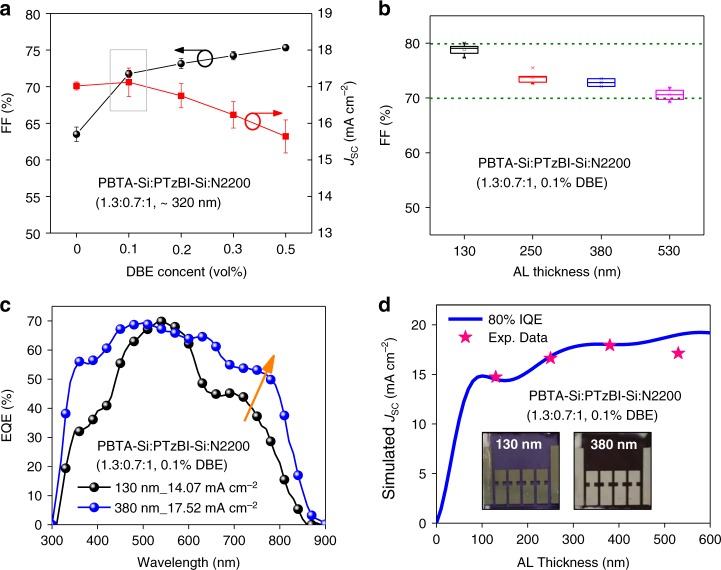
Photovoltaic performance for thick cells based on PBTA-Si:PTzBI-Si:N2200. **a** Dependence of FF and *J*_SC_ on DBE content for 1.3:0.7:1 cells in thick-film condition. **b** FF versus active layer (AL) thickness for 1.3:0.7:1 cells containing 0.1 vol% DBE. **c** EQE response for 1.3:0.7:1-cells with 0.1% DBE; the arrow indicates the EQE increase in NIR region. **d** Simulated *J*_SC_ at 80% IQE and the experimental *J*_SC_ values; insets are photos of 130-nm and 380-nm cell on a 1.5 × 1.5 cm^2^ layout

To optimize the morphology of the BHJ active layer, here we use a non-halogenated dibenzylether (DBE) as the solvent additive (Fig. 1a), which was expected to influence the film-formation dynamics and thus affording time for morphology control^27^. For device based on PBTA-Si:PTzBI-Si:N2200 (1.3:0.7:1) with thickness of about 320 nm, the FF improved continuously with the increased DBE content and maintained at a high level over 70% even when only a trace amount (0.1 vol%) of DBE was incorporated (Fig. 2a). In contrast, the *J*_SC_ decreased gradually when the DBE loading was increased to higher than 0.2 vol% (Supplementary Fig. 9, Supplementary Table 4). As device processed with 0.1 vol% DBE loading gave the highest overall efficiency, we explored the role of DBE in devices with various active layer thickness (Supplementary Fig. 10, Supplementary Table 5). As shown in Fig. 2b, an extremely high FF of 80% was realized for a 130-nm all-PSC, and the FF remained as high as 70% for a much thicker film of 530 nm, which might be correlated to the improved film morphology (will be discussed in the ‘Morphology Investigation’ section).

The 380-nm cell with 0.1 vol% DBE delivered an experimental *J*_SC_ approaching 18 mA cm^−2^, giving a high efficiency of 10.4% on a device area of 0.05 cm^2^ (Table 1). It is worth noting that the 380-nm thick-film device processed with 0.1 vol% DBE shows a much higher EQE response in the N2200-absorbing region than the 130-nm thin-film counterpart (Fig. 2c and Supplementary Fig. 11), agreeing well with the optical simulation analysis. Note that the experimental *J*_SC_ values integrated from the EQE are consistent with the simulated values at a constant IQE of 80% (Fig. 2d), confirming the reliability of the optical modeling. We further simulated the *J*_SC_ based on the experimental IQE (Fig. 1d), which was averaged between 450–650 nm to give a value of 84.5% and 79.3% for all-PSCs with active layer of 110 ± 20 and 350 ± 30 nm, respectively (Supplementary Fig. 12), demonstrating the reliability of using an IQE of 80–90% to estimate the *J*_SC_ for devices with varied active layer thickness. Additionally, the thick-film cell exhibited an excellent thermal stability with the efficiency remaining around 90% of the original value after continuous thermal-annealing at 65 °C for 500 h (Supplementary Fig. 13). Photos of the actual 0.05-cm^2^ cells on a 1.5 × 1.5 cm^2^ layout with a 130-nm and 380-nm active layer are embedded in Fig. 2d. Note that DBE plays a similar role in reference systems of PCE10:N2200 and PBDB-T:N2200 (Supplementary Fig. 15, Supplementary Table 3), confirming the validity of incorporating DBE as additive for processing all-PSCs.

### Morphology investigation

**Fig. 3:**
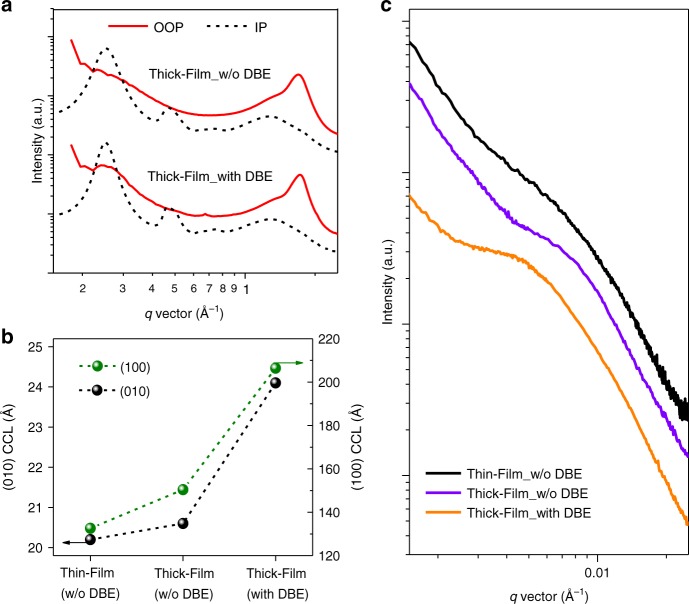
Morphology for PBTA-Si:PTzBI-Si:N2200 (1.3:0.7:1) blend films. **a** GIWAXS line-cuts for thick films without DBE and with 0.1 vol% DBE (OOP: out-of-plane; IP: in-plane). **b** Crystal coherence length (CCL) for the (010) and (100) scattering peaks. **c** RSoXS circular averaged profiles for various 1.3:0.7:1-blend films

The two-dimensional (2D) grazing incidence wide-angle X-ray scattering (GIWAXS) textures indicate that the molecular packing orientation prefers a face-on orientation (Fig. 3a), which is insensitive to film thickness and solvent additive (Supplementary Fig. 16). As the polymer chains have similar lamellae and π–π packing characteristics that are difficult to separate, we fit the (100) and (010) peaks to evaluate the overall crystalline features inside the blend films. It is noted that despite the blend films processed without DBE exhibit similar crystal coherence length (CCL) at different thickness, the thick-film processed with DBE exhibits much higher CCL values in both (100) and (010) diffractions (Fig. 3b). These results suggest a more ordered micro-structure in DBE-processed film, which can inherently suppress recombination loss and lead to high FF in thick film device.

The phase-separation of these films were studied by resonant soft X-ray scattering (RSoXS) under a photon energy of 285.6 eV (Fig. 3c). To minimize the error in determining the scattering peak, we fit the RSoXS profiles by a correlation-length model, *I*(*q*) = A*/q*^n^ + C*/*(*qξ*)^m^ + background, where A*/q*^*n*^ represents Porod scattering from large-sized clusters, and C*/*(*qξ*)^m^ represents a Lorentzian function that depicts scattering from small entities. Here the parameter *ξ* describes the polymer chains’ correlation length, double of which (2*ξ*) can be used to evaluate the domain size when scatter centers are assumed with 50% volume ratio (Supplementary Table 6)^28^. It is noted that the thick film processed with DBE shows an increased domain size of 29 nm relative to that of 21 nm for the film processed without an additive, suggesting better defined domains for the DBE-processed thick film. This is understandable as DBE (with a high boiling point of 298 °C) could offer more time for rearrangement of polymer chains. This is also consistent with the observed denser and longer polymer fibers in thick film processed with DBE shown in atomic force microscopy images (Supplementary Fig. 17). Further studies by combining transmission electron microscopy (TEM) and cross-section energy-dispersive X-ray spectroscopy demonstrated the uniform distribution of three components in the BHJ layer (Supplementary Figs. 18 and 19), implying great promise of utilizing this all-polymer system for fabricating large-area devices.

### Large-area devices

**Fig. 4:**
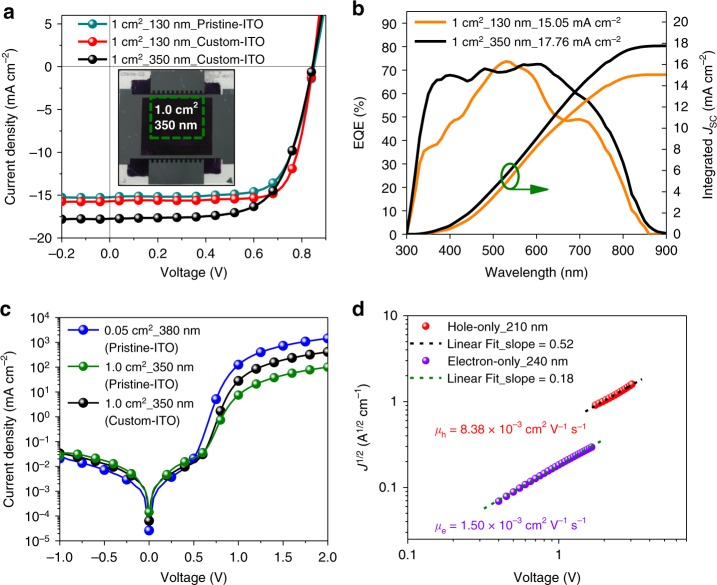
Photovoltaic performance for large-area cells. **a** *J–V* characteristics for 1-cm^2^ cells with different substrate and varied AL thickness, inset is photo of 350-nm cell on a custom-ITO layout (2.5 × 2.5 cm^2^). **b** EQE curves and integrated current density for 1-cm^2^ cells (custom-ITO). **c** Dark *J–V* curves for 0.05-cm^2^ and 1-cm^2^ cells in thick-film condition. **d** *J*^1/2^*-V* characteristics for hole- and electron-only devices

Having established the excellent thickness tolerance for this all-PSC, i.e., the minimal sensitivity of *J*_SC_ and FF regarding to active layer thickness, we further studied the dependence of photovoltaic performance on the device area by enlarging effective area from 0.05 to 1 cm^2^ (see the inset in Fig. 4a). The demonstration of 1 cm^2^-sized solar cell is considered to be one of the critical and challenging steps for up-scaling organic photovoltaic devices towards large-scale production and industrial applications. This is understandable since electrical loss from the bottom ITO electrode, geometric loss induced by increasing cell width, and additional losses caused by film inhomogeneity, defects, or particles will further limit the photovoltaic performance of all-PSCs with enlarged active area^29,30^. For all-PSCs with an active area of 1 cm^2^, the 130-nm device exhibits an impressive PCE of 9.8% with a high FF of about 76% and a *J*_SC_ of 15.05 mA cm^−2^. In contrast, the 350-nm-thick device exhibits a similar PCE of 10.0% with a decreased FF of about 67% while an improved *J*_SC_ of 17.76 mA cm^−2^ (Fig. 4a, Table 1). Note that the *J*_SC_ of the 1-cm^2^ device is slightly higher than the small-area device (0.05 cm^2^), which can be attributable to the higher transmittance of the large-area custom-ITO (Supplementary Fig. 20)^31^. The enhanced *J*_SC_ was consistent with the EQE spectra (Fig. 4b), and the accuracy of *J*_SC_ integrated from the EQE spectra was confirmed by using light-focusing spots in three different locations of the 1.0-cm^2^ cell (Supplementary Fig. 21).

For devices based on pristine ITO, the decreased FF of the 1 cm^2^-devices is correlated to the slightly lower shunt resistance (*R*_P_) of 4.85 × 10^4^ Ω cm^2^, which is lower than that of 8.37 × 10^4^ Ω cm^2^ for the 0.05-cm^2^ device (Supplementary Tables 7 and 8), as calculated from the dark *J*–*V* characteristics shown in Fig. 4c. Here the 1-cm^2^ device was also fabricated based on custom-ITO, which has a metal frame on the peripheral (inset of Fig. 5a) that can compensate for the relatively high sheet resistance (14.6 ± 0.1 Ω/sq) of the central ITO (Supplementary Table 9) and thus give a much lower series resistance (2.2 Ω cm^2^) than that (8.8 Ω cm^2^) of pristine-ITO (Supplementary Table 8). This might account for the much higher FF of 1 cm^2^-devices based on custom-ITO with the same active layer thickness (Supplementary Table 7). It is also worth noting that the 1 cm^2^-devices have a near-square electrode configuration with a large width around 1 cm (1.15 × 0.87 cm^2^, inset of Fig. 5a). Although such large width is essentially unfavorable for efficient charge extraction with respect to the stripe configuration in sub-cells^32^, it is critical in modules where the ratio of cell width to connection width determines the geometric fill factor^33^. Here we employed this challenging cell configuration to demonstrate the potential of active layers that can give minimal efficiency loss during up-scaling process. Furthermore, the high and balanced hole (*μ*_h_ = 8.38 × 10^−3^ cm^2^ V^−1^ s^−1^) and electron (*μ*_e_ = 1.50 × 10^−3^ cm^2^ V^−1^ s^−1^) mobilities measured by space-charge limited current method further rationalize the impressively high FF of these large-area all-PSCs (Fig. 4d, Supplementary Fig. 22).

To demonstrate that the fill factor and overall performance of large-area device are largely determined by the film-quality of active layers, we probed the uniformity of blend films in large-area devices by using the Raman mapping, for which the Raman characteristic of each polymer was plotted to model the component distribution (Supplementary Fig. 23). It is noted that the PTB7-Th:N2200 blend film displays a layered component distribution with a number of pinholes or particles distributed across the entire film. The PBDB-T:N2200 blend film shows a favorable meshy distribution while presenting a few of inhomogeneity near the surface. In contrast, the PBTA-Si:PTzBI-Si:N2200 blend presents a fine-divided network continuously dispersed across the entire region for both PBTA-Si:PTzBI-Si and N2200 film, indicating the high-quality of this blend film with minimal inhomogeneity (Supplementary Fig. 24). Such high quality and uniform film are consistent with the minimal decay of photovoltaic performance, in particular the FF, upon the scale-up to large-area device, which is of critical importance toward practical applications.

## Discussion

This work demonstrates the impressive absorption behavior of the most widely used polymer acceptor N2200, and reports a strategy to improve the external quantum efficiency response in the NIR region for NDI-based all-PSCs. Optical simulation reveals that the conventional active layer thickness of around 100 nm is too thin for efficient light absorption by N2200, while the thick-film of over 300 nm leads to severe recombination that cause significantly decreased fill factor and reduced short-circuit current density. Here the trade-off between light absorption and charge recombination is successfully addressed by integrating a ternary siloxane-based all-polymer system with mixed-ether solvent processing. Combined film morphology characterizations clearly elucidate the mechanism of suppressed charge recombination induced by DBE treatment.

In view of the realization of efficient large-area devices based on imide-functionalized benzotriazole (TzBI)-polymers, we emphasize the importance of excellent solubility and good film-forming property of photoactive materials for reducing the efficiency loss in large-area cells. Moreover, considering the semi-crystalline characteristic of TzBI-polymers and the shared structure segment of imide in PTzBI-Si and N2200, it is rational to suppose that the relatively flexible skeleton of polymers and sufficient molecular intermixing between different species are also favorable for obtaining high-quality film and thus relieving the efficiency losses. These correlations facilitate further understanding of the design principles of photoactive materials for high-performance large-area solar cells with minimal up-scaling losses.

In summary, we successfully reconcile two urgent but conflicting issues in NDI-based all-PSCs, i.e., insufficient light harvesting in the acceptor-absorbed region and severe charge recombination in thick-film devices, by virtue of managing the light propagation and tuning the blend-film micro-morphology. Through optical simulation, we find that a thick bulk film of approximately 350 nm is needed to promote the *J*_SC_ above 20 mA cm^−2^. Combined with a morphology-optimization strategy using the non-halogenic additive DBE, we demonstrate a very high FF reaching 80% for small-area (0.05 cm^2^) devices under thin-film (130 nm) condition, and an experimental *J*_SC_ approaching 18 mA cm^−2^ together with a high FF over 72% for a 380-nm-thick cell, leading to an impressively high PCE of 10.4%. More importantly, a promising efficiency over 10% is maintained for all-PSCs on a device area of 1 cm^2^, which mitigates the gap relative to that in organic solar cells based on small molecular acceptors.

## Methods

### Materials

PTzBI-Si, PBTA-Si, PFNDI-Br, and N2200 were synthesized in our own lab according to the previous reported methods^34,35^. PCE10 and PBDB-T were purchased from 1-Material Inc. without further purification.

### Fabrication and characterization of solar cells

The device structures were indium tin oxide (ITO)/PEDOT:PSS (Clevios P VP Al 4083, 40 nm)/active layer/PFNDI-Br (5 nm)/Ag, where PEDOT:PSS and PFNDI-Br represent poly(3,4-ethylenedioxythiophene):polystyrene sulfonate and poly[(9,9-bis(3′-((*N*,*N*-dimethyl)-*N*-ethylammonium)propyl)-2,7-fluorene)-*alt*-5,5′-bis(2,2′-thiophene)-2,6-naphthalene-1,4,5,8-tetracaboxylic-*N*,*N*′-di(2-ethylhexyl)imide]dibromide, respectively. ITO glasses were treated with 2-min oxygen plasma before 40 nm PEDOT:PSS being spun-cast at 4000 rpm. The substrates were dried at 150 °C/20 min in air and then transferred into a nitrogen-protected glove box. PBTA-Si:PTzBI-Si:N2200 blends were dissolved in MeTHF at 100 °C for 1 h before coating. The total donor concentrations were set as 4.0 and 5.5 mg mL^−1^ to get thin (1200 rpm, 130 ± 20 nm) and thick (900 rpm, 350 ± 30 nm) films, respectively. For those incorporating additive, certain content of DBE was added 30 min before coating. All these blend films were annealed at 130 °C for 10 min, and those with DBE were then stored in low vacuum for at least 2 h. The PCE10:N2200 (1:1) and PBDB-T:N2200 (1:1) blends were dissolved in CB at 60 °C overnight before coating. For PCE10:N2200, donor concentration of 6.0 mg mL^−1^ and 9.5 mg mL^−1^ were used to form a 110 nm and 320 nm film, respectively, while for PBDB-T:N2200, 4.0 mg mL^−1^ and 8.0 mg mL^−1^ were needed to obtain a film of 80 nm and 260 nm, respectively. PFNDI-Br (0.5 mg mL^−1^) in MeOH or MeOH:EtOH (1:1) was then spin-coated onto the active layer at 2000 rpm. Then silver electrode (about 100 nm) was thermally deposited on the top of the PFNDI-Br layer under vacuum (5 × 10^−7^ torr) in the presence of a shadow mask (opening area: 0.0516 cm^2^ or 1.0 cm^2^). The effective device area was further defined based on a non-refractive mask (which was determined to be 0.04 cm^2^ or 0.9062 cm^2^) to ensure the accuracy of the experiments. The characteristics of current density (*J*) as a function of voltage (*V*) were recorded on a Keithley 2400 source measurement unit, and the measurement was carried out under AM 1.5 G (1 sun) based on a class AAA solar simulator (Enlitech SS-F5). The simulated sun-light intensity (100 mW cm^−2^) was calibrated by using a standard silicon solar cell (certified by NREL). The light path for the outlet area is 16 cm^2^. The EQE spectra were recorded by using a commercially available QE-R measurement system (Enlitech Inc.) with a spot size of 0.01 cm^2^.

### UV–vis absorption and reflection measurements

The measurement of UV–vis absorption spectra was carried out by using a SHIMADZU UV-3600 spectrophotometer. The reflection (R) was measured based on a commercially available QE system (QE-R, Enlitech Inc.) by using an integrating sphere, calibrated by a white reflective sheet. The film samples were prepared on a substrate of glass/Ag (90 nm). The real absorption of active layer was determined from 1-R, with the background of bare glass/Ag (90 nm) deducted.

### Optical simulation

The measurement of optical parameters (*n* and *k*) was carried out by using a dual rotating-compensator ME-L ellipsometer (Wuhan Eoptics Tech.). Optical simulation was performed by calculating the one-dimensional distribution of the optical-electromagnetic field of the device from the transfer-matrix formalism (TMF). The IQE was assumed as a constant in the entire range of wavelength, and the modeling *J*_SC_ was estimated by multiplying the IQE value with the number of photons harvested in the active layer.

### SCLC measurements

The *J–V* characteristics of hole-only (ITO/PEDOT:PSS/active layer/MoO_3_/Ag) and electron-only (ITO/ZnO/active layer/PFNDI-Br/Ag) devices were recorded by using a Keithley 236 sourcemeter. The measurements were carried out under dark. The hole mobility and electron mobility of the active layer were obtained by fitting quadratic SCLC region using the equation of *J* = (9/8)ε_0_ε_r_*μV*^*2*^/*d*^3^, where *µ* is the zero-filed mobility,ε_0_ represents the permittivity of free space, and ε_r_ represents the relative permittivity. The effective voltage was determined by subtracting the applied voltage (*V*_appl_) with the voltage drop *(V*_s_) and the built-in voltage (*V*_bi_).

### Film thickness and AFM measurements

Active layer thickness was recorded on a surface profiling system (DektakXT, Bruker). The samples were prepared on a substrate of ITO/PEDOT:PSS (40 nm), therefore the single active layer thickness was obtained as the total thickness (*T*_total_) minus 40 nm. AFM topographic morphologies were recorded by using a Bruker Multimode 8 Microscope (tapping-mode). Thin-film (about 130 nm) and thick-film (about 350 nm) samples were deposited on the top of the PEDOT:PSS-coated ITO through spin-casting method.

### GIWAXS and RSoXS measurements

GIWAXS characterization was performed at the beamline 7.3.3 of Advanced Light Sourceat, Lawrence Berkeley National Laboratory (LBNL). The X-ray beam energy of this beamline was 10 keV, operating at top-off mode. The sample to detector distance was around 273 mm which was calibrated by a silver behenate. All the samples were spin-coated on the top of the PEDOT:PSS/silicon wafer substrates. The incidence angle was set to be 0.16°. The scattering signal of each sample was recorded with a 5 s exposure time on a 2D charge-coupled device (CCD) detector (Pilatus 2 M) with a pixel size of 0.172 × 0.172 mm^2^. The measurement of RSoXS was carried out at beamline 11.0.1.2 at the Advanced Light Source, LBNL. Sample preparation was the same as that of GIWAXS samples. The polymer blend films were floated in deionized water and transferred atop silicon nitrile windows, and dried in air. By screening the beam energies from 280 to 290 eV, the scattering signals were collected in vacuum by using a Princeton Instrument PI-MTE CCD camera (pixel size of 0.027 × 0.027 mm^2^).

### EDS and TEM measurements

EDS measurement was conducted by using an analytical electron microscope with atomic-resolution (FEI Titan Themis 200). TEM cross-section images were recorded from a JEM 2100 F Microscope. The sample preparation process is exactly the same as solar cell device fabrication, with a normal structure and a thick active layer of about 260 nm.

### Raman measurements

Raman spectra and maps were recorded under ambient condition by using a Raman spectrometer (Renishaw inVia Reflex, with maximum power of 50 mW; with a 532 nm DPSS laser). The measurements were carried out by using a long focal length objective (50 ×), with NA of 0.5 (Leica N Plan L 50×), and a grating with 2400 lines mm^−1^.

### Reporting summary

Further information on research design is available in the Nature Research Reporting Summary linked to this article.

## Supplementary information

Supplementary Information

Reporting Summary

## Data Availability

The relevant data are available from the authors upon reasonable request.
