# Supplementary material for: Surpassing the 10% efficiency milestone for 1-cm2 all-polymer solar cells
Source: Nat Commun. 2019 Sep 10;10:4100. doi: 10.1038/s41467-019-12132-6 (PMC6736853; doi:10.1038/s41467-019-12132-6)
Supplement: Supplementary file 1 — Supplementary Information [file 41467_2019_12132_MOESM1_ESM.pdf]

SUPPLEMENTARY INFORMATION

Surpassing the 10% efficiency milestone for 1-cm^2 all-polymer solar cells

Fan et al.

SUPPLEMENTARY FIGURES

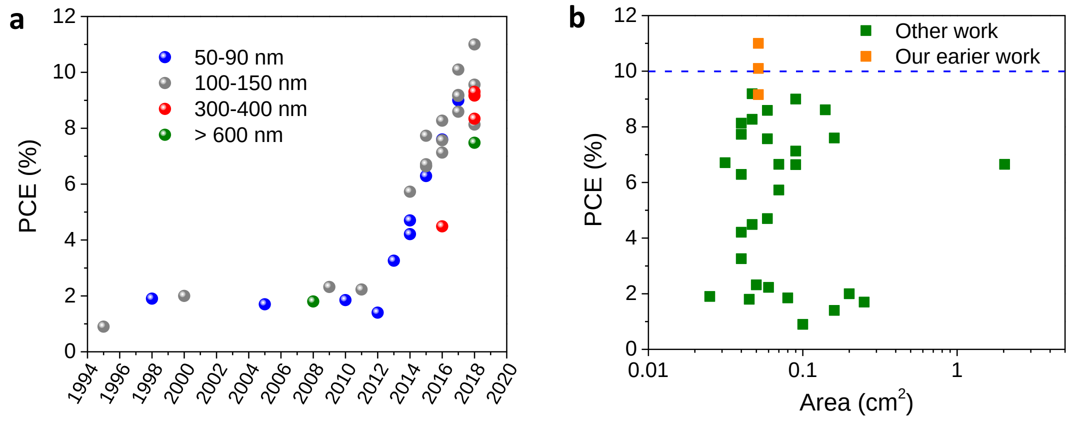

Supplementary Figure 1. Statistics of all-PSCs. a, Historical performance progress for small-area (<0.05 cm^2) all-PSCs with various thickness scales. **b,** Efficiency of all-PSCs reported thus far on different device areas.

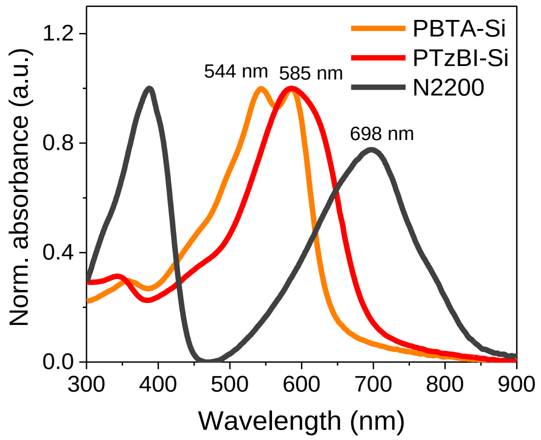

Supplementary Figure 2. Normalized absorbance for neat films of PBTA-Si, PTzBI-Si, and N2200.

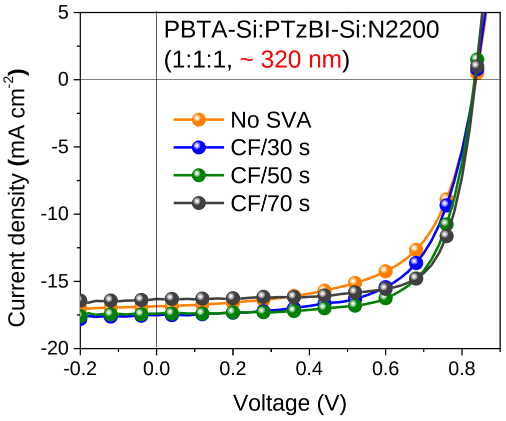

Supplementary Figure 3. *J-V* characteristics for thick all-PSCs (~320 nm) based on PBTA-Si:PTzBI-Si:N2200 (1:1:1) with CF annealing for varied time.

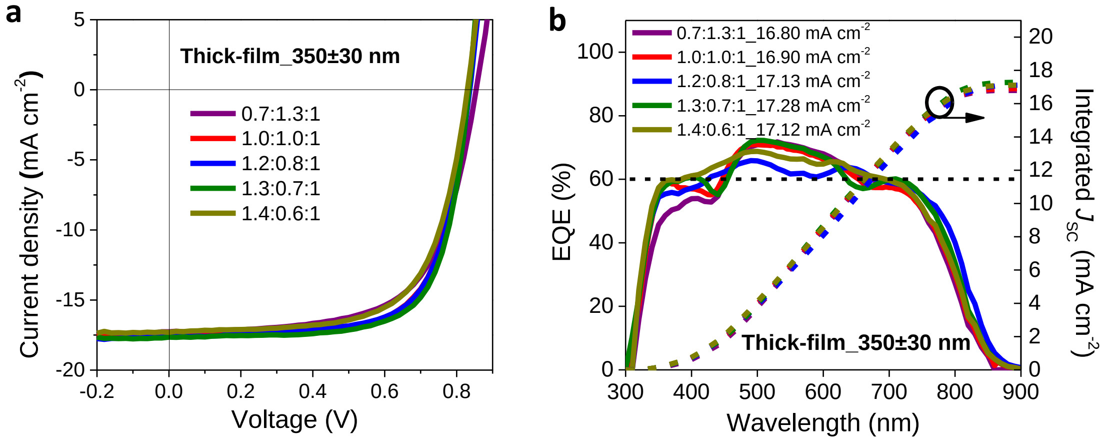

Supplementary Figure 4. *J-V* characteristic (**a**) and EQE response (**b**) for all-PSCs based on PBTA-Si:PTzBI-Si:N2200 with varied blending ratios in thick-film condition.

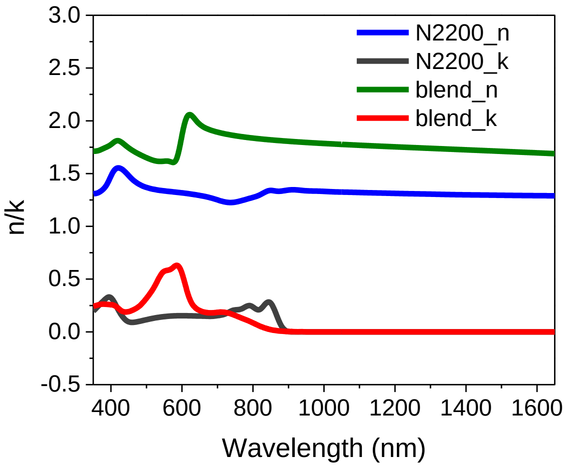

Supplementary Figure 5. The dependence of refractive index (*n*) and extinction coefficient (*k*) on the wavelength for both N2200 neat film and 1.3:0.7:1-blend film.

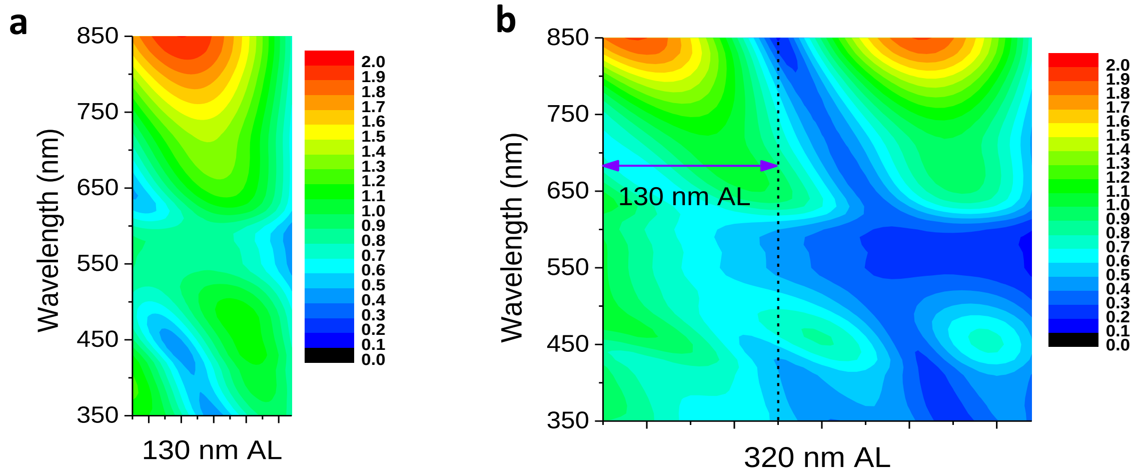

Supplementary Figure 6. Distribution of optical electromagnetic field E^2 for 1.3:0.7:1 cells with active layer thickness of 130 nm (**a**) and 320 nm (**b**), simulated by transfer matrix formalism, with the device structure of glass (0.7 mm)/ITO (180 nm)/PEDOT:PSS (40 nm)/active layer (AL)/PFNDI-Br (5 nm)/Ag (90 nm); the color bar represents the electric field intensity.

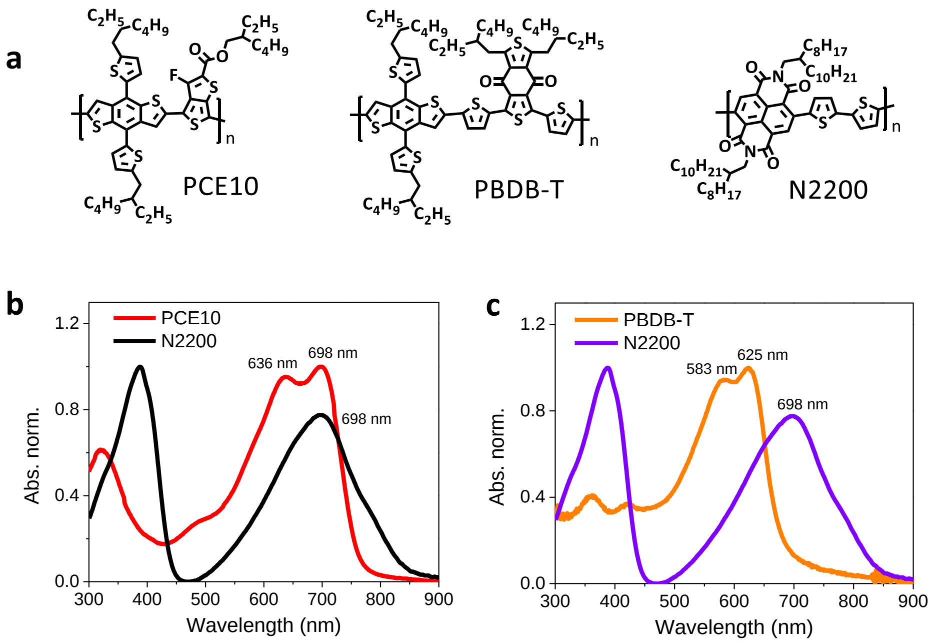

Supplementary Figure 7. Absorption profiles. a, Chemical structure of active layer components. **b,** Normalized absorption for neat films of PTB7-Th and N2200. **c,** Normalized absorption for neat films of PBDB-T and N2200.

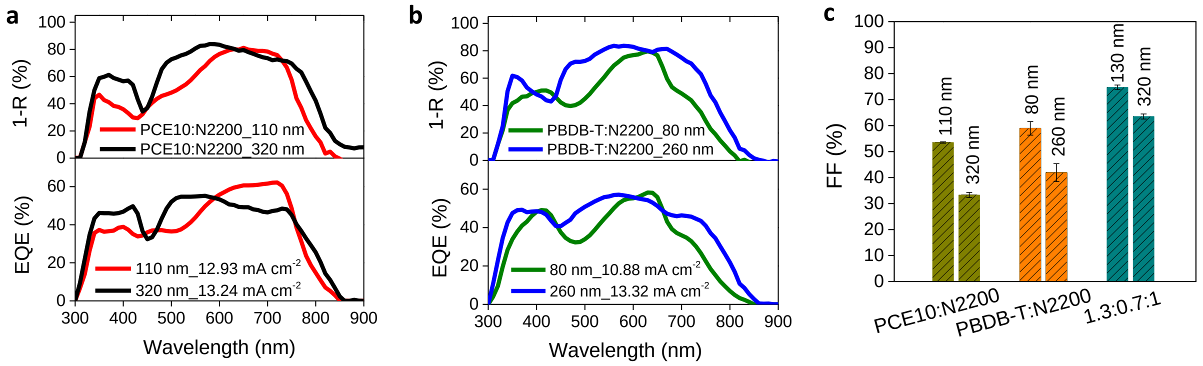

Supplementary Figure 8. Performance for PCE10:N2200 and PBDB-T:N2200 systems. a-b, Device absorption (1-R) and EQE spectra for blends of PCE10:N2200 (**a**) and PBDB-T:N2200 (**b**) with different thickness. **c**, FF decay with increased active layer (AL) thickness for all-PSCs based on various systems, where PBTA-Si:PTzBI-Si:N2200 (1.3:0.7:1) system is depicted as reference.

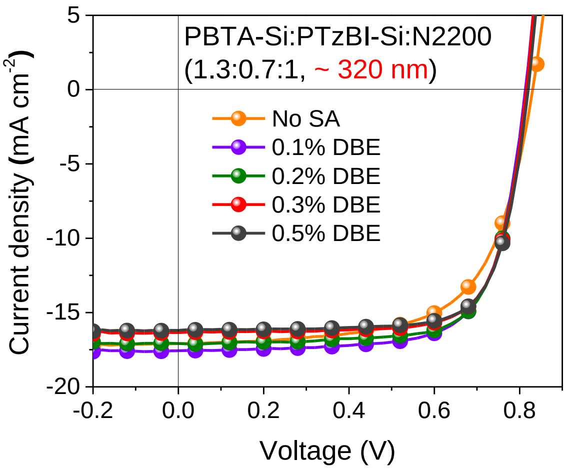

Supplementary Figure 9. *J-V* characteristics for thick all-PSCs (~320 nm) based on PBTA-Si:PTzBI-Si:N2200 (1.3:0.7:1) incorporating various content of DBE additive.

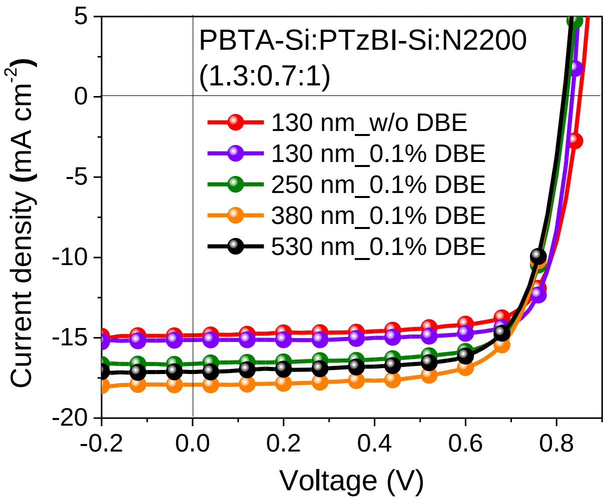

Supplementary Figure 10. *J*-*V* characteristics for all-PSCs (area = 0.05 cm^2) based on PBTA-Si:PTzBI-Si:N2200 (1.3:0.7:1) with 0.1vol% DBE incorporated.

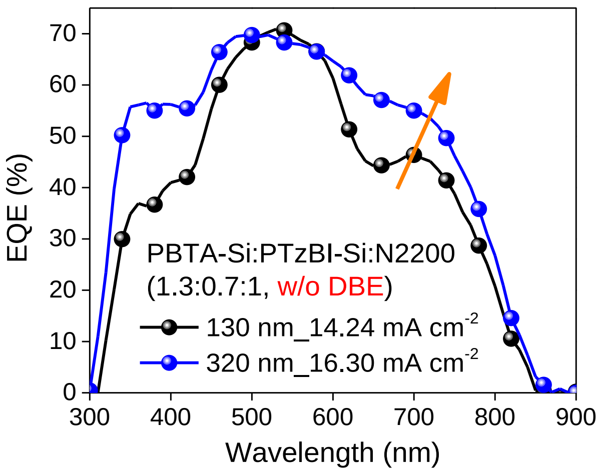

Supplementary Figure 11. EQE response for all-PSCs based on PBTA-Si:PTzBI-Si:N2200 (1.3:0.7:1) without additive; the arrow indicates the EQE increase in NIR regime.

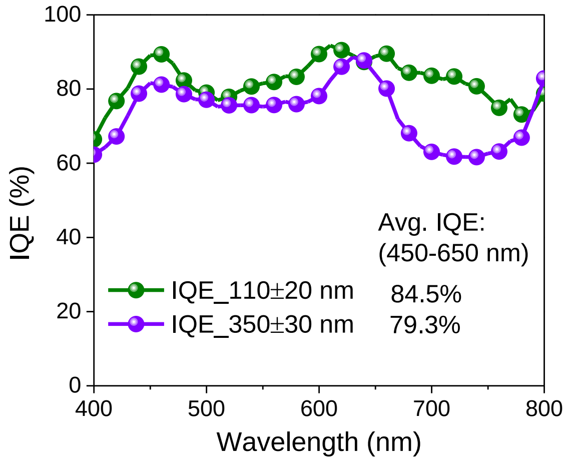

Supplementary Figure 12. The IQE spectra for all-PSCs with active layer around 110 nm and 350 nm.

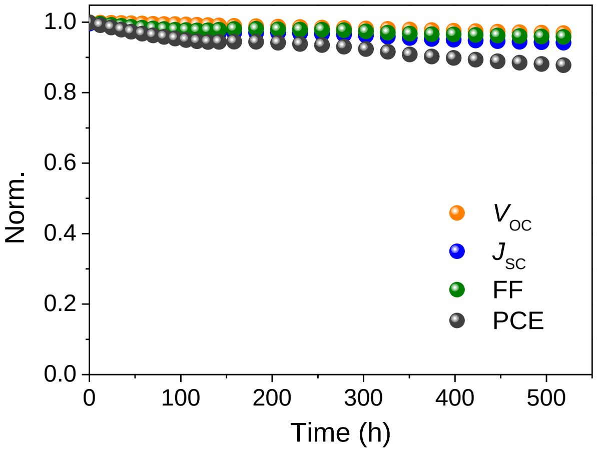

Supplementary Figure 13. Thermal stability for the thick-film cell after continuously thermal-annealed at an operating temperature of 65 ^{o}C for >500 h.

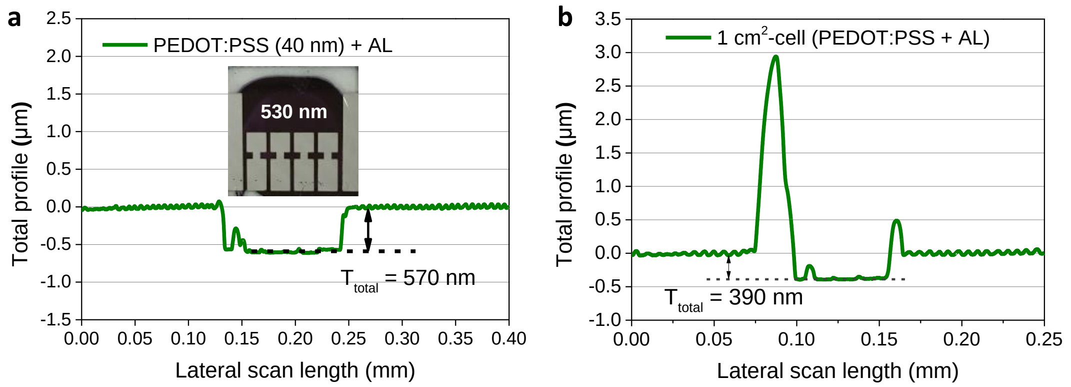

Supplementary Figure 14. Thickness distribution for thick all-PSCs with area of 0.05 cm^2 (**a**) and 1.0 cm^2 (**b**) measured by step profiler. The inset is the photo of 530 nm-cell on a 1.5×1.5 cm^2 layout.

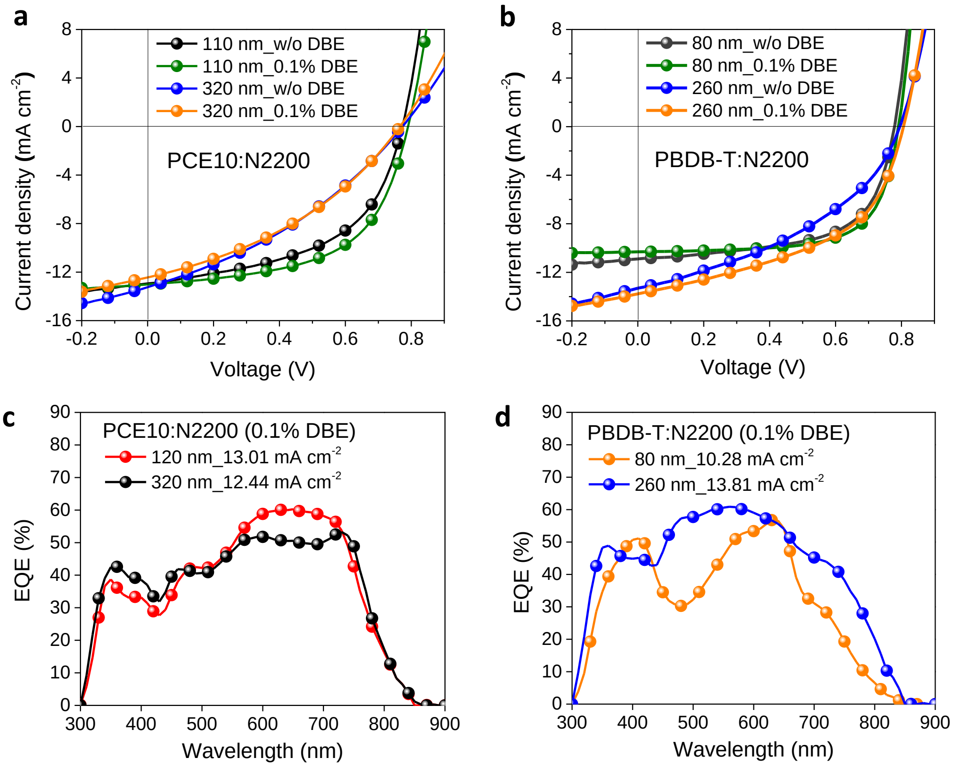

Supplementary Figure 15. *J-V* characteristic (**a, b**) and EQE response (**c, d**) for all-PSCs based on PCE10:N2200 (**a, c**) and PBDB-T:N2200 (**b, d**), respectively.

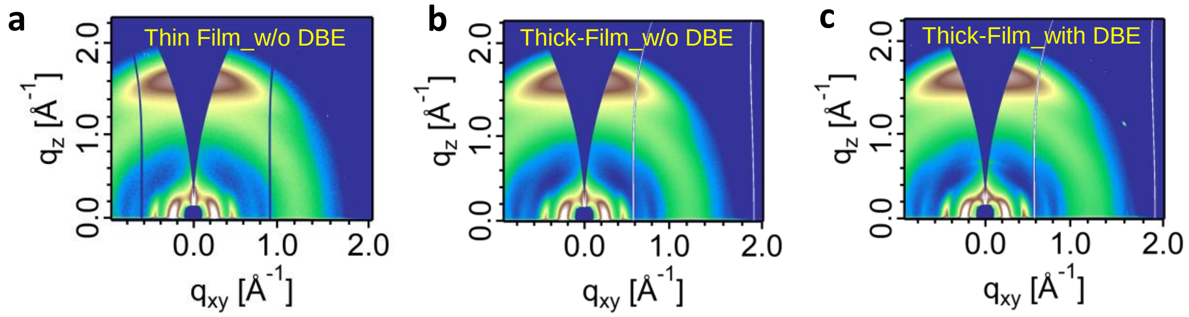

Supplementary Figure 16. 2D GIWAXS patterns for thin film without DBE (**a**), and for thick films without DBE (**b**) and with 0.1 vol% DBE (**c**).

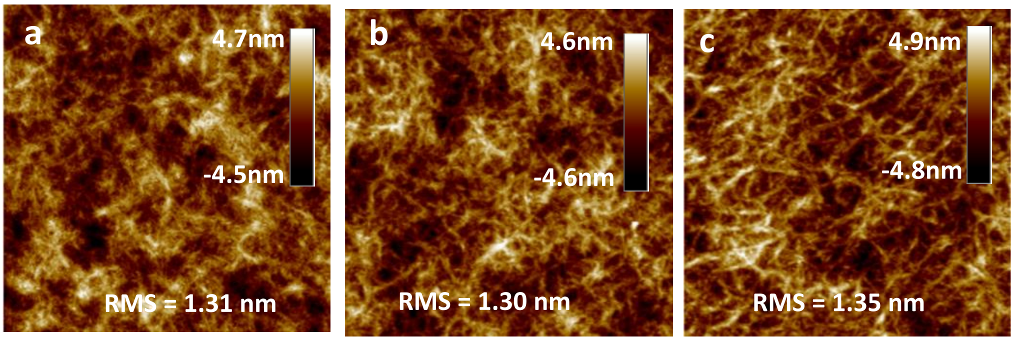

Supplementary Figure 17. AFM images (2×2 μm^2) for PBTA-Si:PTzBI-Si:N2200 (1.3:0.7:1) blend films. a-c, AFM for thin-film without DBE (**a**), thick-film without DBE (**b**), and thick-film with 0.1vol% DBE (**c**).

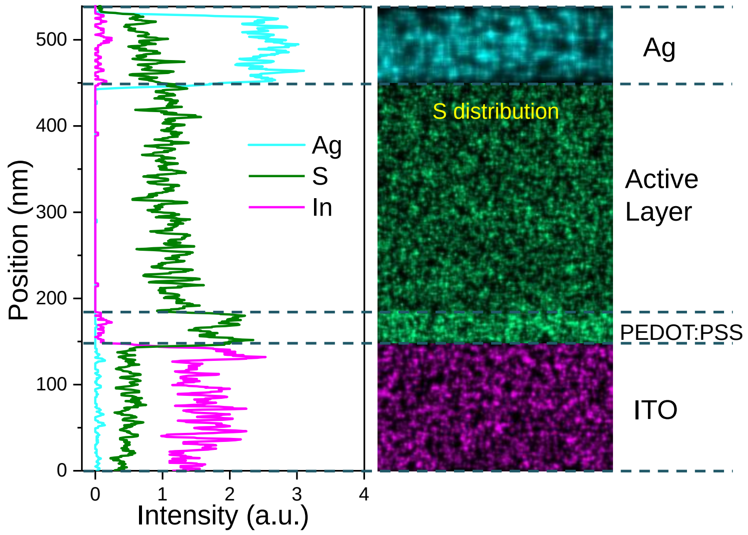

Supplementary Figure 18. Sulfur distribution in PBTA-Si:PTzBI-Si:N2200-based device with a structure of ITO (180 nm)/PEDOT:PSS (30 nm)/active layer (260 nm)/PFNDI-Br (5 nm)/Ag (90 nm), measured by energy-dispersive X-ray spectroscopy (EDS).

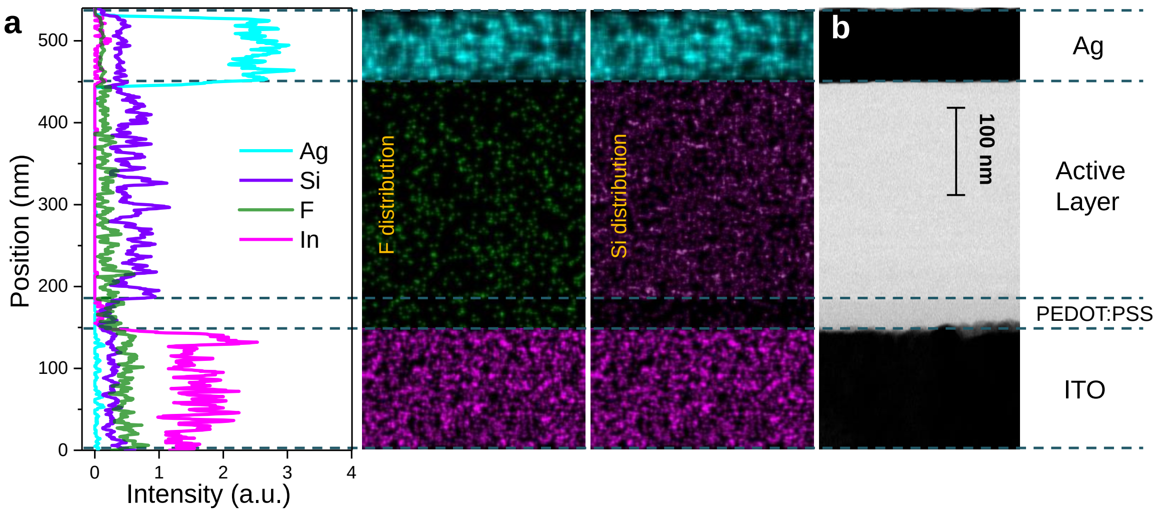

Supplementary Figure 19 | Vertical distribution of Si and F. EDS (**a**) and cross-section TEM (**b**) for PBTA-Si:PTzBI-Si:N2200-based device with a structure of ITO (180 nm)/PEDOT:PSS (30 nm)/active layer (260 nm)/PFNDI-Br (5 nm)/Ag (90 nm).

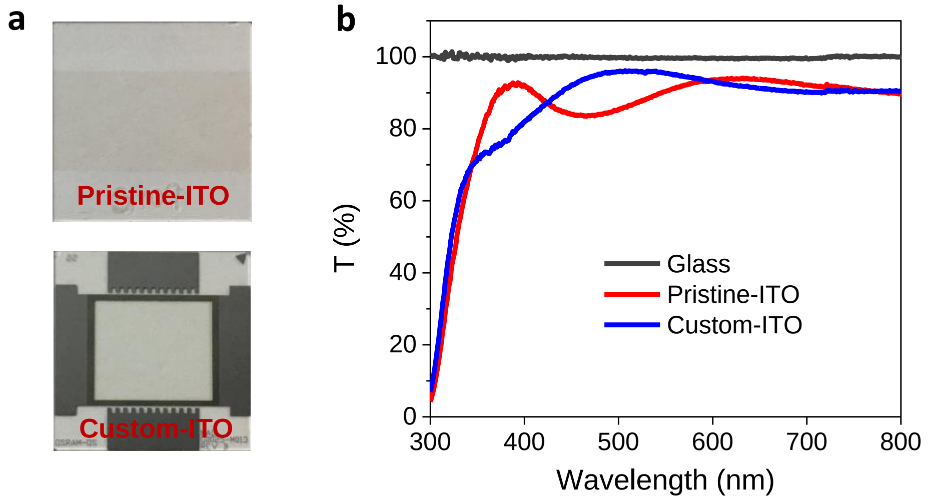

Supplementary Figure 20. Photos and transmittance for pristine-ITO and custom-ITO with glass as reference.

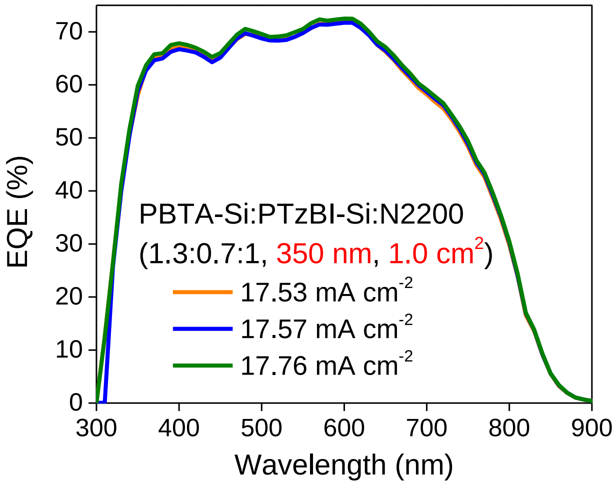

Supplementary Figure 21. EQE curves for all-PSCs based on PBTA-Si:PTzBI-Si:N2200 (1.3:0.7:1), with light spots focusing on three different locations of 1.0 cm^2-cell.

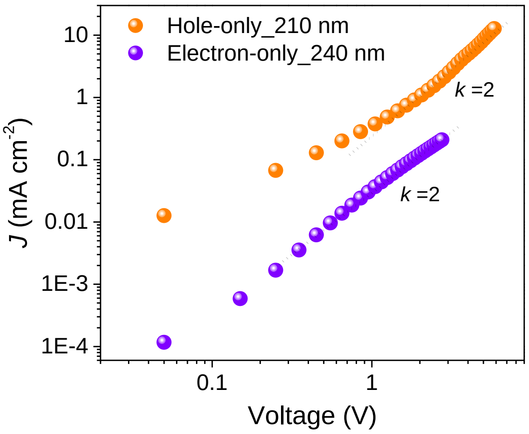

Supplementary Figure 22. *J-V* characteristics for hole- and electron-only devices under the single-carrier device structure of ITO/PEDOT:PSS/active layer/MoO_3/Ag and ITO/ZnO/ active layer/PFNDI-Br/Ag, respectively.

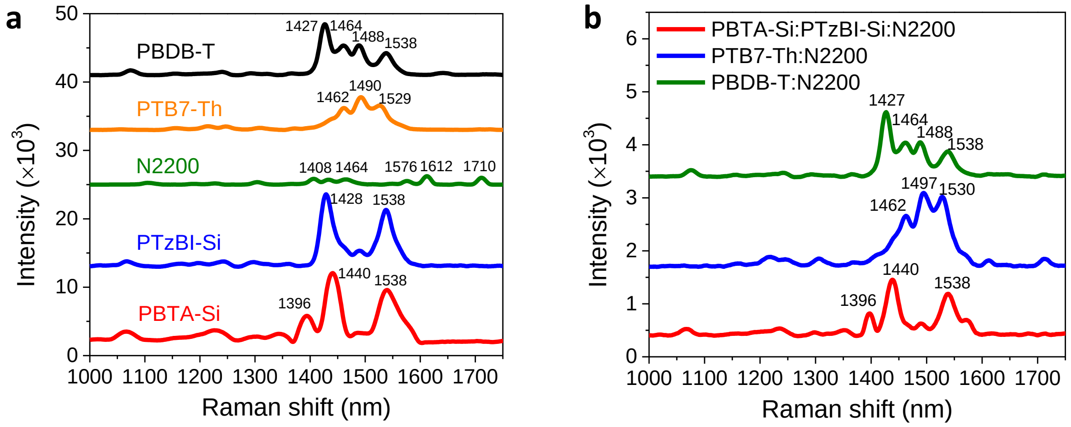

Supplementary Figure 23. a, Raman spectra for neat polymers as powder. **b,** Raman spectra for polymer blends as films.

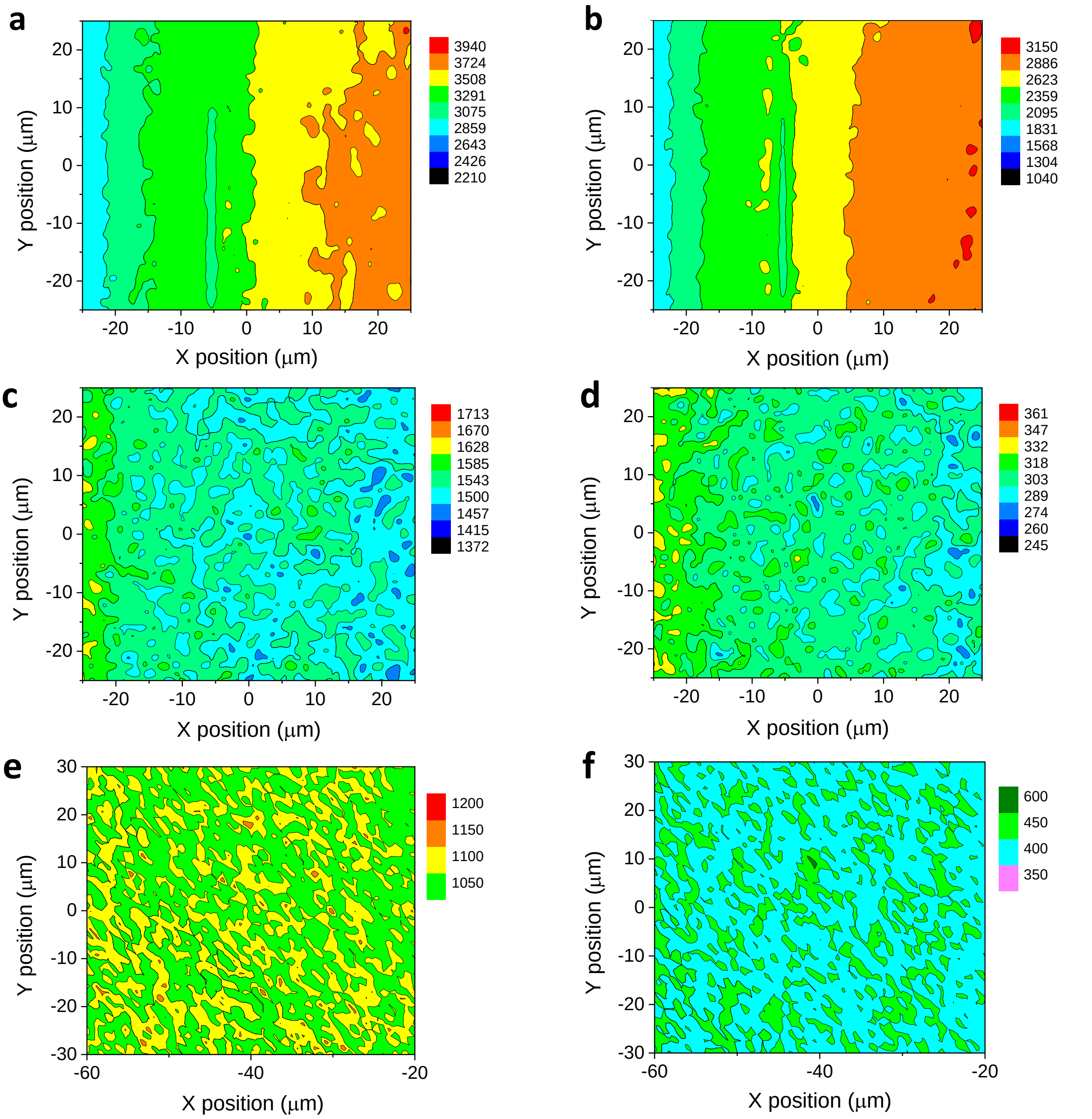

Supplementary Figure 24. Raman mapping (50×50 μm^2 or 60×40 μm^2) for blend film of PTB7-Th:N2200 (1:1, ~320 nm), PBDB-T:N2200 (1:1, ~260 nm), and PBTA-Si:PTzBI-Si:N2200 (1.3:0.7:1, ~320 nm). a, Distribution of PTB7-Th detected from the peak at 1497 nm. **b,** Distribution of N2200 detected from the peak at 1612 nm. The color bar represents the Raman intensity. **c,** Distribution of PBDB-T detected from the peak at 1427 nm. **d,** Distribution of N2200 detected from the peak at 1612 nm. The color bar represents the Raman intensity. **e,** Distribution of PBTA-Si:PTzBI-Si detected from the peak at 1440 nm. **f,** Distribution of N2200 detected from the peak at 1612 nm. The color bar represents the Raman intensity.

SUPPLEMENTARY TABLES

Supplementary Table 1. Photovoltaic parameters for thick all-PSCs (~320 nm) based on PBTA-Si:PTzBI-Si:N2200 (1:1:1) by CF annealing.

Blend ratio	CF annealing (V)	V_{OC} (V)	J_{SC} (mA cm^{-2})	FF (%)	PCE (%)
1:1:1	/	0.837	16.85	61.73	8.70
	30 s	0.835	17.52	64.38	9.42
	50 s	0.833	17.40	69.73	10.11
	70 s	0.836	16.30	73.90	10.07

Supplementary Table 2. Photovoltaic parameters for thick all-PSCs (350±30 nm) based on PBTA-Si:PTzBI-Si:N2200 with various blending ratios; the device structure is ITO/PEDOT:PSS/active layer (CF-SVA/50 s)/PFNDI-Br/Ag.

Blend ratio	V_{OC} (V)	J_{SC} (mA cm^{-2})	$J_{SC, EQE}$ (mA cm^{-2})	FF (%)	PCE (%)
0.7:1.3:1	0.854	17.27	16.80	64.38	9.50
1.0:1.0:1	0.833	17.40	16.90	69.73	10.11
1.2:0.8:1	0.837	17.60	17.13	68.82	10.14
1.3:0.7:1	0.834	17.65	17.28	71.02	10.45
1.4:0.6:1	0.830	17.30	17.12	66.26	9.51

Supplementary Table 3. Photovoltaic parameters for all-PSCs based on PCE10:N2200 and PBDB-T:N2200; the device structure is ITO/PEDOT:PSS/active layer/PFNDI-Br/Ag.

Blend	Thickness (nm)	Additive	V_{OC} (V)	$J_{SC, EQE}$ (mA cm^{-2})	FF (%)	PCE [a] (%)
PCE10:N2200	110	/	0.77	12.93	51.90	5.17
		0.1% DBE	0.79	13.01	56.92	5.85
	320	/	0.79	13.24	34.73	3.56
		0.1% DBE	0.77	12.44	37.12	3.56
PBDB-T:N2200	80	/	0.78	10.88	61.21	5.19
		0.1% DBE	0.79	10.28	68.28	5.55
	260	/	0.80	13.32	40.38	4.28
		0.1% DBE	0.81	13.81	48.44	5.42

[a] The efficiency was obtained by using the calculated current density form EQE.

Supplementary Table 4. Photovoltaic parameters for thick all-PSCs (~320 nm) based on PBTA-Si:PTzBI-Si:N2200 (1.3:0.7:1) incorporating various content of DBE additive.

DBE (vol%)	V_{OC} (V)	J_{SC} (mA cm^{-2})	FF (%)	PCE (%)
/	0.830	17.09 (17.02±0.11)	64.63 (63.50±0.98)	9.17
0.1	0.813	17.57 (17.12±0.39)	71.28 (71.75±0.49)	10.19
0.2	0.816	17.08 (16.75±0.33)	72.90 (73.15±0.70)	10.16
0.3	0.816	16.35 (16.23±0.36)	74.39 (74.24±0.58)	9.92
0.5	0.820	16.20 (15.64±0.45)	74.68 (75.32±0.42)	9.92

Supplementary Table 5. Photovoltaic parameters for all-PSCs (area = 0.05 cm^2) based on PBTA-Si:PTzBI-Si:N2200 (1.3:0.7:1) with 0.1vol% DBE incorporated.

Thickness (nm)	V_{OC} (V)	J_{SC} (mA cm^{-2})	FF (%)	PCE (%)
130	0.831 (0.832±0.003)	14.74 (14.64±0.40)	80.02 (79.15±0.72)	9.80 (9.64±0.30)
250	0.822 (0.818±0.005)	16.63 (16.48±0.59)	73.82 (73.76±1.13)	10.09 (9.93±0.24)
380	0.815 (0.818±0.003)	17.93 (17.63±0.42)	72.10 (72.81±1.01)	10.54 (10.49±0.06)
530	0.816 (0.814±0.003)	17.13 (17.24±0.39)	71.87 (70.58±1.10)	10.05 (9.90±0.13)

Supplementary Table 6. Domain spacing, crystal coherence length (CCL), and correlation length extracted from GIWAXS and RSoXS.

Sample	*d* spacing[a] (100) [Å]	CCL (100)[a] [Å]	*d* spacing[a] (010) [Å]	CCL (010)[a] [Å]	Correlation Length[b] [Å]	Domain Size[b] [nm]
Thin_w/o DBE	24.7	132.6	3.7	20.2	130	26
Thick_w/o DBE	24.8	150.4	3.7	20.6	105	21
Thick_w DBE	24.9	206.4	3.7	24.1	145	29

[a]Data extracted from GIWAXS. [b]Data obtained from RSoXS fitting.

Supplementary Table 7. Photovoltaic parameters for large-area all-PSCs based on PBTA-Si:PTzBI-Si:N2200 (1.3:0.7:1, 0.1% DBE) with different ITO substrates.

Area (cm^2)	Substrate	V_{OC} (V)	J_{SC} (mA cm^{-2})	FF (%)	PCE (%)	Thickness (nm)
1.0	Pristine-ITO	0.850	15.24	71.29	9.24	130
		0.847	17.15	59.24	8.61	350
	Custom-ITO	0.846	15.72	76.29	10.15	130
		0.844	17.76	66.78	10.01	350

Supplementary Table 8. Series resistance (R_S), shunt resistance (R_P) for devices based on PBTA-Si:PTzBI-Si:N2200 (1.3:0.7:1), obtained from dark *J*–*V* characteristics.

Area (cm^2)	R_S[a] (Ω cm^2)	R_P[a] (Ω cm^2)
0.05 (Pristine-ITO)	0.7	8.37×10^4
1.0 (Pristine-ITO)	8.8	4.85×10^4
1.0 (Custom-ITO)	2.2	4.94×10^4

[a] R_S and R_P were calculated around 2 V and 0 V, respectively, from the dark *J*–*V* characteristics.

Supplementary Table 9. Sheet resistance of various ITO substrates, measured by linear four-probe square resistance tester.

Substrate	R_{sq}[a)] (Ω/sq)	Avg. R_{sq} (Ω/sq)
Pristine-ITO	7.9/8.0/8.1	8.0±0.1
Custom-ITO	14.7/14.5/14.6	14.6±0.1

[a)] R_{sq} was calculated from the formule, $R_{sq} = 4.5324\ \Delta V_{23}/I_{14}$, where I_{14} is the current flowing through the probe 1 and 4, and ΔV_{23} represents the potential difference between probe 2 and 3.
