# Supplementary material for: Surpassing the 10% efficiency milestone for 1-cm2 all-polymer solar cells
Source: Nat Commun. 2019 Sep 10;10:4100. doi: 10.1038/s41467-019-12132-6 (PMC6736853; doi:10.1038/s41467-019-12132-6)
Supplement: Supplementary file 2 — Reporting Summary [file 41467_2019_12132_MOESM2_ESM.pdf]

nature research

Corresponding author(s): Lei Ying, Ning Li, Fei Huang

Solar Cells Reporting Summary

Nature Research wishes to improve the reproducibility of the work that we publish. This form is intended for publication with all accepted papers reporting the characterization of photovoltaic devices and provides structure for consistency and transparency in reporting. Some list items might not apply to an individual manuscript, but all fields must be completed for clarity.

For further information on Nature Research policies, including our data availability policy, see Authors & Referees.

Experimental design

Please check: are the following details reported in the manuscript?

1. Dimensions

Area of the tested solar cells

☒ Yes — Large-area cells: 1.0 cm2; small-area cells:0.05 cm2.
☐ No — *Explain why this information is not reported/not relevant.*

Method used to determine the device area

☒ Yes — Image measuring instrument.
☐ No — *Explain why this information is not reported/not relevant.*

2. Current-voltage characterization

Current density-voltage (J-V) plots in both forward and backward direction

☐ Yes — *State where this information can be found in the text.*
☒ No — we have demonstrated that the scan direction has little influence on the I-V curves of organic solar cells based on PTzBI-derivatives.

Voltage scan conditions
For instance: scan direction, speed, dwell times

☒ Yes — We used a dwell time of 0.2 s for each voltage and a step voltage of 0.02 V.
☐ No — *Explain why this information is not reported/not relevant.*

Test environment
For instance: characterization temperature, in air or in glove box

☒ Yes — The cells were measured in grove box with the temperature fixed at ~25 °C during the I-V scans.
☐ No — *Explain why this information is not reported/not relevant.*

Protocol for preconditioning of the device before its characterization

☒ Yes — No preconditioning protocol like encapsulation was used before characterization.
☐ No — *Explain why this information is not reported/not relevant.*

Stability of the J-V characteristic
Verified with time evolution of the maximum power point or with the photocurrent at maximum power point; see ref. 7 for details.

☐ Yes — *State where this information can be found in the text.*
☒ No — N.A.

3. Hysteresis or any other unusual behaviour

Description of the unusual behaviour observed during the characterization

☐ Yes — *State where this information can be found in the text.*
☒ No — No hysteresis or any other unusual behaviors were observed in the testing.

Related experimental data

☐ Yes — *State where this information can be found in the text.*
☒ No — N.A.

4. Efficiency

External quantum efficiency (EQE) or incident photons to current efficiency (IPCE)

☒ Yes — We provided EQE spectra for various cells in Figure 2c, Figure 4b, Supplementary Figure 4b, Supplementary Figure 8, Supplementary Figure 11, and Supplementary Figure 15c, d, and Supplementary Figure 21.
☐ No — *Explain why this information is not reported/not relevant.*

A comparison between the integrated response under the standard reference spectrum and the response measure under the simulator

☒ Yes — We provided the comparison between the integrated Jsc from the EQE and the Jsc obtained from I-V scan in Table 1.
☐ No — *Explain why this information is not reported/not relevant.*

For tandem solar cells, the bias illumination and bias voltage used for each subcell

☐ Yes — *State where this information can be found in the text.*
☒ No — N.A.

5. Calibration

Light source and reference cell or sensor used for the characterization

☒ Yes ☐ No

A class AAA solar simulator (Taiwan, Enlitech SS-F5) was used as light source, providing 100 mW cm-2 of simulated AM I.5G irradiation, which was calibrated by a standard silicon solar cell (certified by NREL).

Explain why this information is not reported/not relevant.

Confirmation that the reference cell was calibrated and certified

☒ Yes ☐ No

The reference standard silicon solar cell was certified by NREL.

Explain why this information is not reported/not relevant.

Calculation of spectral mismatch between the reference cell and the devices under test

☐ Yes ☒ No

N.A.

N.A.

6. Mask/aperture

Size of the mask/aperture used during testing

☒ Yes ☐ No

The aperture area of 0.9062 cm2 and 0.04 cm2 were used for testing cells with area of 1.0 cm2 and 0.05 cm2, respectively.

Explain why this information is not reported/not relevant.

Variation of the measured short-circuit current density with the mask/aperture area

☐ Yes ☒ No

State where this information can be found in the text.

No evident Jsc variation was found in this work.

7. Performance certification

Identity of the independent certification laboratory that confirmed the photovoltaic performance

☐ Yes ☒ No

State where this information can be found in the text.

We did not provide the certification of photovoltaic performance, but the aperture area we used has been certificated by National Institute of Metrology (NIM) in Beijing, China.

A copy of any certificate(s)
Provide in Supplementary Information

☐ Yes ☒ No

State where this information can be found in the text.

N.A.

8. Statistics

Number of solar cells tested

☒ Yes ☐ No

We tested at least 16 cells for each type of devices, with the standard error shown in Table 1.

Explain why this information is not reported/not relevant.

Statistical analysis of the device performance

☐ Yes ☒ No

State where this information can be found in the text.

N.A.

9. Long-term stability analysis

Type of analysis, bias conditions and environmental conditions
For instance: illumination type, temperature, atmosphere humidity, encapsulation method, preconditioning temperature

☒ Yes ☐ No

Long-term thermal stability was provided in Supplementary Figure 13.

N.A.
